# Vaginally Administered PEGylated LIF Antagonist Blocked Embryo Implantation and Eliminated Non-Target Effects on Bone in Mice

**DOI:** 10.1371/journal.pone.0019665

**Published:** 2011-05-18

**Authors:** Ellen Menkhorst, Jian-Guo Zhang, Natalie A. Sims, Phillip O. Morgan, Priscilla Soo, Ingrid J. Poulton, Donald Metcalf, Estella Alexandrou, Melissa Gresle, Lois A. Salamonsen, Helmut Butzkueven, Nicos A. Nicola, Evdokia Dimitriadis

**Affiliations:** 1 Embryo Implantation, Prince Henry's Institute, Clayton, Australia; 2 Cancer and Haematology, The Walter and Eliza Hall Institute, Parkville, Australia; 3 Bone, Joint and Cancer, St Vincent's Institute of Medical Research, Melbourne, Australia; 4 Multiple Sclerosis, Howard Florey Institute, Melbourne, Australia; 5 Department of Medicine, The University of Melbourne, Parkville, Australia; 6 Endometrial Remodelling, Prince Henry's Institute, Clayton, Australia; Agency for Science, Technology and Research - Singapore Immunology Network, Singapore

## Abstract

Female-controlled contraception/HIV prevention is critical to address health issues associated with gender inequality. Therefore, a contraceptive which can be administered in tandem with a microbicide to inhibit sexually transmitted infections, is desirable. Uterine leukemia inhibitory factor (LIF) is obligatory for blastocyst implantation in mice and associated with infertility in women. We aimed to determine whether a PEGylated LIF inhibitor (PEGLA) was an effective contraceptive following vaginal delivery and to identify non-uterine targets of PEGLA in mice.

Vaginally-applied ^125^I-PEGLA accumulated in blood more slowly (30 min vs 10 min) and showed reduced tissue and blood retention (24 h vs 96 h) compared to intraperitoneal injection in mice. Vaginally-applied PEGLA blocked implantation. PEGLA administered by intraperitoneal injection inhibited bone remodelling whereas vaginally-applied PEGLA had no effect on bone. Further, PEGLA had no effect in an animal model of multiple sclerosis, experimental auto-immune encephalomyelitis, suggesting PEGLA cannot target the central nervous system.

Vaginally-administered PEGLA is a promising non-hormonal contraceptive, one which could be delivered alone, or in tandem with a microbicide. Vaginal application reduced the total dose of PEGLA required to block implantation and eliminated the systemic effect on bone, showing the vagina is a promising site of administration for larger drugs which target organs within the reproductive tract.

## Introduction

The World Health Organization has called for the urgent development of pharmacological, non-hormonal contraceptives [1]. More than 700,000 maternal deaths, most in the developing world and related to causes associated with unintended pregnancies, occurred between 1995 and 2000; more than 400,000 of these deaths resulted from unsafe abortions [2]. Safe, affordable and reliable contraception improves maternal and child health and reduces population growth [3], which will also help to reduce the consequences of climate change [4]. It is estimated that over 200 million women worldwide want, but currently lack, access to modern contraceptives [4]. Female controlled contraception/HIV prevention is critical to address health issues associated with gender inequality [5]. Progress in the contraceptive development arena has been so poor that a recent report by the United Kingdom All Party Parliamentary Group on Population, Development and Reproductive Health [6] concluded that the Millennium Development Goals of the United Nations cannot be met given the levels of population growth in the poorest countries.

Implantation of a blastocyst into the uterine endometrium is a critical step for the establishment of pregnancy. Synchronized endometrial receptivity and blastocyst competence is essential for implantation and is achieved via a regulated network of paracrine and autocrine factors, including cytokines [7].

Leukemia inhibitory factor (LIF), an interleukin (IL) 6-type cytokine, is one of the few molecules obligatory for fertility in mice [8]. LIF null female mice are infertile due to the failure of blastocysts to implant into the uterus [8]. In women, LIF production by the uterine epithelium is maximal during the period of ‘uterine receptivity’ [9], [10], [11], a short window during the menstrual cycle when the uterus is capable of responding to and allowing blastocyst implantation [7]. Evidence for an important role of LIF in human implantation comes from clinical studies of infertile women, who have lower levels of *LIF* mRNA and protein in endometrial tissue and LIF protein in uterine flushings than fertile women [12], [13], [14], [15]. In vitro, exogenous LIF enhances the adhesion of primary human endometrial epithelial cells to fibronectin [16], an extracellular matrix component present on trophoectodermal cells of the blastocyst [17] and to collagen IV [16], present on first trimester human trophoblast [18]. Altogether these studies suggest that LIF modulates adhesion between endometrial epithelial and trophoblast cells. We hypothesise that blockage of LIF action in women would prevent blastocyst implantation.

In mice, interperitoneal (IP) injections of a highly potent, PEGylated (conjugated to polyethylene glycol) LIF antagonist (PEGLA) during the peri-implantation period blocks endometrial epithelial LIF action and prevents blastocyst implantation [19], making PEGLA a promising pharmacological contraceptive. PEGLA antagonises LIF by binding to the LIF receptor (R) but not recruiting the LIFR signalling component, IL6ST (also known as gp130), preventing initiation of downstream gene transcription [20]. Blastocysts recovered from PEGLA-treated females outgrow normally in culture [19], showing that PEGLA acts only on the endometrium in mice.

The LIFR is also utilised for signalling by other IL6 family members including oncostatin M (OSM), ciliary neurotrophic factor (CNTF) and cardiotrophin 1 (CT-1). The LIF^−/−^ mouse is infertile due to failure of implantation [8] and shows decreased bone volume associated with increased osteoclast number and size [21]. The LIFR^−/−^ mouse is embryonic lethal and shows impaired placental formation, decreased bone volume associated with increased osteoclast number and size, decreased sensory neuron survival, decreased numbers of spinal and brainstem astrocytes and elevated liver glycogen [22]. While CT-1 knockout mice have normal fertility, neonate CT-1 null mice show a similar phenotype to the LIF^−/−^ and LIFR^−/−^ mouse [23]. Currently no information is available as to the non-uterine tissue targets of PEGLA; however low bone volume in LIF^−/−^, CT-1^−/−^ and LIFR^−/−^ mice [21], [22] and reduced numbers of spinal and brainstem astrocytes in LIFR^−/−^ mice suggests that PEGLA may also influence bone structure and astrocyte number.

In women, vaginally administered drugs preferentially localise to the uterus [24], [25]. This preferential localisation, termed the ‘uterine first-pass effect’, was identified because of the marked uterine response to vaginally administered progesterone despite low serum progesterone concentrations [24]. Subsequent studies showed that the endometrial concentration of vaginally administered drugs was substantially higher than following other routes of delivery, including intramuscular and oral [26], [27], [28]. The mechanism of preferential localisation to the uterus is not clear, although it seems likely that drugs administered via the vagina are absorbed into veins within the upper third of the vagina and then transported by counter-current exchange to the uterine arteries [25], [29], [30].

In women, vaginal application has been used as a delivery method for contraceptives that primarily target the ovary [31], [32] although these are anti-progestins which are very small. PEGLA is estimated to be ∼60 kDa. It is not known whether such a large molecule can be absorbed by the vagina to enter the blood and reach the uterus. To the best of our knowledge, the efficacy of vaginally applied contraceptives that target the uterus has not been reported. Numerous vaginally delivered microbicides, which inhibit sexually transmitted infections (STI), including HIV, are currently undergoing clinical trials. Recently, a clinical trial of using a vaginal gel formulation of tenofovir, a nucleotide reverse transcriptase inhibitor, showed significant inhibition of HIV incidence [33], suggesting that tenofovir could be vaginally administered in tandem with a contraceptive such as PEGLA, making a ‘dual-role’ contraceptive, capable of preventing both pregnancy and STIs.

We hypothesized that PEGLA would localise to the uterus more rapidly and at a higher concentration following vaginal delivery than following injection, making vaginal application a promising contraceptive delivery method for PEGLA in women. Our specific aims were to: 1. establish the contraceptive efficacy of PEGLA following vaginal delivery in mice; 2. determine whether bone and the central nervous system (CNS) are non-uterine targets of PEGLA in mice and 3. to compare the tissue distribution and half-life of PEGLA following IP injection and vaginal application in mice.

## Materials and Methods

### Animals

C57BL6/J mice (Monash Animal Services, Clayton, WEHI Bioservices Department, Kew, both VIC, Australia) housed under conventional conditions, had ad libitium food and water and were maintained in a 12 h: light-dark cycle. All procedures were approved by the Monash Medical Centre (B) Animal Ethics Committee (#MMCB2007/21) or the WEHI Animal Ethics Committee (#2008.032), and this study followed the NHMRC Australian Code of Practice for the Care and Use of Animals for Scientific Purposes.

### Preparation of PEGylated proteins

PEGLA was produced as previously described [19], except that Y-NHS-40K (Jenkem Technology, Allen, Texas, USA) was used for PEGylation.

The in vitro activity of PEGLA was measured using a LIF-responsive Ba/F3 line stably expressing human LIFR and IL6ST as previously described [19]. PEGLA produced with the new PEG compound exhibited a 2-fold increase in the inhibition of LIF-induced proliferation of Ba/F3 cells [20] compared to the original PEGLA (Figure S1).

Control PEGylation reagent was generated by incubating Y-NHS-40K in Milli-Q water for at least 24 h. PEGylated bovine serum albumin (BSA) or mouse serum albumin (MSA; both Sigma-Aldrich, Castle Hill, NSW, Australia) were prepared as for PEGLA except that an anion exchange step was performed.

### Vaginal application of PEGLA

#### Tissue localization of PEGLA

Mated female mice (8 weeks; n = 2/group) were given a single vaginal application of PEGLA (11.25 µg/g [250 mM]/application) dissolved 1∶2 in a placebo gel (CAPRISA 400 Study Gel, provided by the Consortium for Industrial Collaboration in Contraceptive Research Program [CICCR] of the Contraceptive Research and Development Program [CONRAD]) at 9am on day (D) 3 (day of vaginal plug detection = D0) and killed after 3 h or 24 h or were given multiple doses (×10) of 3.75 µg/g [83.3 mM]/application PEGLA during D2–5 and killed on D6. Uteri were dissected out and fixed in Neutral Buffered Formalin before PEGLA immunoreactivity in the vagina and uterus was identified as previously described [34].

#### Contraceptive efficacy of PEGLA

Mated female mice (8–10 weeks; n = 4/group) were given vaginal applications of PEGLA at two doses (4.8 µg/g [111 mM] or 5.6 µg/g [111 mM]/application) or PEGMSA control (4.8 µg/g [111 mM] or 5.6 µg/g [111 mM]/application) dissolved 1∶2 in placebo gel during the peri-implantation period. The mouse vagina can hold only ∼15 µl, thus the dose of PEGLA able to be delivered per application was limited. Mated female mice were given a total of 5 applications at D2 9am, 3pm & 9pm and D3 9am & 3pm. It was not possible to treat mice with a greater number of applications as this resulted in abortion in the SHAM treated mice (data not shown). Treated mice were killed on D6 and the number of CL on the ovary and IS in the uterus counted.

#### The effect of PEGLA on long-term fertility

Non-mated female mice (10 weeks; n = 3/group) were given IP injections of PEGLA (24.9 µg/g [81.5 mM]/injection) or PEGylation reagent control (PEG; 20 µg/g [100 mM]/injection) at 12pm and 10pm and the following day at 10am. After 15 days the treated mice were paired until they plugged. Plugged mice were killed on D10 and the number of implantation sites in the uterus counted.

#### The effect of PEGLA on bone remodelling

Mated and non-mated female mice (8–10 weeks; n = 4/group) were treated with PEGLA by IP injection or vaginal application. IP injections of PEGLA (30 µg/g [100 mM]/injection) or PEGylation reagent control (PEG; 20 µg/g [100 mM]/injection) were given to mated and non-mated females at 12pm and 10pm (D2 in mated females) and the following day at 10am (D3 in mated females). Vaginal applications of PEGLA (5 µg/g [111 mM]/application) or PEGMSA (5 µg/g [111 mM]/application) were given to mated females at D2 9am, 3pm & 9pm and D3 9am & 3pm).

Treated mice were killed on D6 and tibiae analysed as previously described [35].

#### Effect of PEGLA on EAE

In mice, LIF and CNTF prevent worsening of experimental auto-immune encephalomyelitis (EAE), an inducible animal model of multiple sclerosis [36], [37]. We investigated whether PEGLA could inhibit LIF action in the CNS and worsen EAE. EAE was induced in male mice (8–10 weeks; n = 8/group) using MOG 35–55 (MEVGWYRSPFSRVVHLYRNGK) peptide ([38], Mimitopes). MOG 35–55 (final concentration 0.5 mg/ml) was delivered in equal parts peptide (carried in MT-PBS) and Freund's Complete Adjuvant (Difco) containing *Mycobacterium tuberculosis* H37Ra (4 mg/ml; Difco). Each mouse received a total dose of 125 µg peptide, via 100 µl subcutaneous injections in each flank, as well as a subcutaneous injection of 50 µl in the base of the tail. On the initial day of EAE induction (designated as D0) and D3, the mice also received an IP injection of 300 ng of Bordetella Pertussis toxin (List Biological).

Mice that reached EAE grade 2 (see below), were given water and powdered food in small Petri dishes at the base of the cage for easy access. Cotton wool buds were also added to cages for extra warmth. To monitor general health, mice were weighed every second day. Mice that were considered to be in poor health or that had lost their righting reflex were euthanized by administration of 100 µl lethabarb (325 mg/mL pentobarbitone sodium, Virbac, Peakhurst, NSW) via IP injection.

Disease severity was graded by a blinded observer using a standard EAE scoring system:

0 – No clinical symptoms

1 – Mild tail weakness

1.5 – Mild tail weakness; mild gait abnormality (splayed or high-stepped)

2 – Complete tail atonia

2.25 – Complete tail atonia; mild gait abnormality

2.5 – Complete tail atonia; moderate gait abnormality (trunk cannot be raised)

2.75 – Complete tail atonia; paralysis of one hind limb

3 – Complete tail atonia; paralysis of both hind limbs

3.5 – Complete hind limb and tail paralysis plus animal unable to right when placed supine; animals were euthanized at this point

4 – Death

#### PEGLA Treatment

Mice received IP injections of PEGLA (5 µg/g [16.7 mM]/injection) or PEG control (3.3 µg/g [16.5 mM]/injection) every second day post-induction of EAE.

#### Serum pNF-H Assay

Fresh blood samples (n = 6–7/group), collected by cardiac puncture after euthanasia and just prior to transcardial perfusion, were centrifuged at 5000 rpm for 10 min to separate serum for analysis in the pNF-H ELISA as previously described [39].

### Tissue distribution of ^125^I-PEGLA following IP injection and vaginal application

Proteins were radioiodinated as described previously [40]. Non-pregnant or mated (D2) female mice (n = 3/group) were given ^125^I-PEGLA or control (^125^I-PEGBSA) by IP injection (25 µg/g [83.3 mM] PEGLA plus 4×10^6^ cpm ^125^I-PEGLA, dissolved in sterile saline, total volume: 100 µl) or by vaginal application (15 µg/g [333.3 mM] PEGLA plus 7×10^5^ cpm ^125^I-PEGLA, carried 1∶2 in placebo gel, total volume: 15 µl). At selected times post-application of the radioligand (10 min, 30 min, 2 h, 6 h, 24 h and IP only: 48 h, 72 h, 96 h and 120 h), urine was collected by bladder palpitation, blood collected by cardiac puncture and stored in EDTA tubes (Microvette, Sarstedt, Technology Park, SA, Australia) to prevent clotting, and intact organs weighed and total ^125^I measured in each using a gamma counter (Packard Cobra Multi-detector, Packard, Downers Grove, IL).

Protein-bound ^125^I (^125^I-PEGLA or ^125^I-PEGBSA) in blood was measured by mixing 100 µl blood with 1 ml of 20% (w/v) trichloroacetic acid (TCA, Sigma) on ice for 5 min before samples were spun at 8,000× *g* for 1 min. TCA-precipitated ^125^I was defined as protein-bound ^125^I whereas non-precipitable ^125^I was considered free-iodine. The ^125^I content in intact organs was expressed as counts/mg and in blood and urine as counts/µl. To compare between IP and vaginally applied PEGLA, the ^125^I content was expressed as counts/applied dose (AD) per mg tissue or µl blood/urine.

### Statistics

GraphPad Prisim 5.0 (GraphPad Software, San Diego, CA) was used for all statistical analyses, except for the EAE study which used SigmaStat version 2.03 (SPSS Inc). A *P* value of ≤0.05 was considered statistically significant. A Mann-Whitney t-test was used to compare IS and CL numbers between treatment groups, ^125^I-PEGLA and ^125^I-PEGBSA levels in non-mated animals at 24 h after vaginal application, ^125^I-PEGLA levels between IP injection and vaginal application at each time-point, bone parameters between PEGLA and control mice and EAE grades. Paired t-tests were used to analyse the data from the pNF-H ELISA. A Kruskal-Wallis one-way ANOVA with Dunn's post-hoc test was used to identify differences in ^125^I counts between time-points and to compare ^125^I counts between ^125^I-PEGLA (mated and non-mated) and ^125^I-PEGBSA (non-mated) treated animals at 2 h and 24 h. The pNF-H concentrations were correlated to the EAE grades using a Pearson correlation.

## Results

### Vaginally administered PEGLA localized to the uterine epithelium and blocked implantation

Vaginally applied PEGLA localised to the uterine luminal epithelium and basal surface of the glandular epithelium at 3 h and was similarly present at 24 h (Figure S2A–D). Importantly, the vaginal epithelium remained intact following both single and repeated (×10) applications of PEGLA (Figure S2E–F).

**Figure 1:**
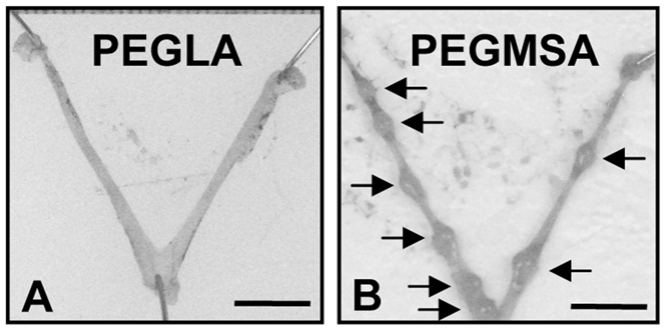
PEGLA blocked implantation following vaginal delivery. Ex vivo uteri following treatment with 5 applications of 5.6 µg/g/application PEGLA (**A**) or PEGMSA (**B)** on day 6. Arrows indicate implantation sites. Bars = 1.0 cm.

Vaginal administration of the higher dose of PEGLA (5 applications; 5.6 µg/g/application) on D2 and D3 blocked implantation (n = 4/group; PEGLA 0/4 females had implantation sites (IS), 0.0 IS/uterus, average body weight 17.8±0.3 g; PEGMSA: 4/4 females had IS, 6.5±1.3 IS/uterus, average body weight 18.0±0.4 g; Figure 1A&B). There was no difference in the number of corpora lutea (CL) between the PEGLA and PEGMSA control treatment groups indicating that normal ovulations had occurred (PEGLA 6.5±0.6; PEGMSA 8.5±0.9 CL/mouse; *P* = 0.1367). By comparison, vaginal administration at the lower dose (4.8 µg/g/application) of PEGLA did not block implantation (n = 4/group; PEGLA 4/4 females had implantation sites (IS), 10.3±0.3 IS/uterus, average body weight 21.5±0.5 g; PEGMSA: 4/4 females had IS, 8.8±0.8 IS/uterus, average body weight 21.8±0.5 g; no difference in implantation site number between PEGLA and PEGMSA treated females: *P* = 0.1367). Histology of uteri from PEGLA treated females showed the uteri were non-pregnant and intact (data not shown) as found previously following IP injection of PEGLA [19].

### PEGLA had no effect on subsequent fertility

All non-pregnant females administered PEGLA or PEG by IP injection mated within 3 days of pairing. No difference in the number of implantation sites was found on D10 (PEGLA 9.3±1.5; PEG 8.0±1.0 IS/uterus; *P* = 0.3687) indicating that normal implantation had occurred.

### PEGLA inhibited bone remodelling following IP injection

**Figure 2:**
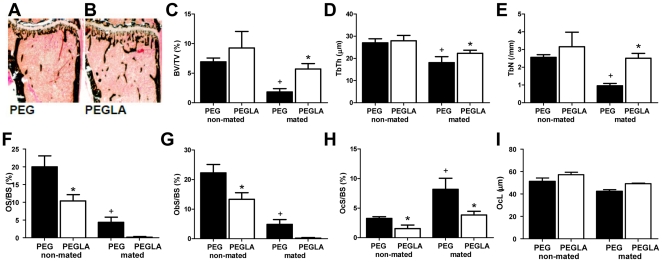
Intraperitoneal injections of PEGLA reduced bone remodelling in mated and non-mated mice. **A & B.** Representative Von Kossa stained tibial sections from mated (**A**) PEGylation reagent and (**B**) PEGLA treated mice show reduced trabecular bone (bone stains black). **C–I.** Tibial (**C**) trabecular bone volume (BV/TV), (**D**) trabecular thickness (TbTh), (**E**) trabecular number (TbN), (**F**) osteoid surface (OS/BS), (**G**) osteoblast surface (ObS/BS), (**H**) osteoclast surface (OcS/BS) and (**I**) osteoclast length (OcL). All values are mean ± SEM for 4–7 mice per group. *, significant difference (*P*<0.05) to PEGylation reagent controls of the same mating status; +, significant difference (*P*<0.05) to non-mated PEGylation reagent controls.

Histomorphometry of the tibia following 3 IP injections of PEGLA (30 µg/g/injection) to both mated and non-mated females showed that PEGLA increased trabecular bone volume, trabecular number and trabecular thickness (Figure 2A–E) in mated females when compared to PEG treated controls. Interestingly, mated females had a lower amount of trabecular bone (Figure 2C–E) than non-mated females on D6 regardless of treatment. Non-mated females treated with PEGLA had less osteoid (Figure 2F) and fewer osteoblasts (Figure 2G) and osteoclasts (Figure 2H) than control females. Mated females treated with PEGLA also had fewer (Figure 2H) osteoclasts than control females, but osteoblast and osteoid surface were not significantly lowered (Figure 2F&G) possibly because the level of osteoblast and osteoid in mated females was already low compared to non-mated females (Figure 2F&G).

### PEGLA had no effect on bone following vaginal administration

**Figure 3:**
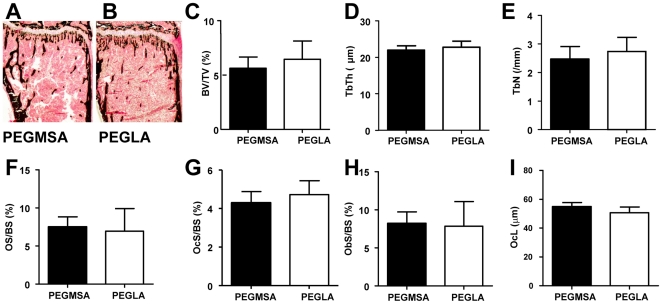
Vaginal applications of PEGLA had no effect on bone remodelling in mated mice. **A & B.** Representative Von Kossa stained tibial sections from mated (**A**) PEGMSA and (**B**) PEGLA treated mice show reduced trabecular bone (bone stains black). **C–I.** Tibial (**C**) trabecular bone volume (BV/TV), (**D**) trabecular thickness (TbTh), (**E**) trabecular number (TbN), (**F**) osteoid surface (OS/BS), (**G**) osteoblast surface (ObS/BS), (**H**) osteoclast surface (OcS/BS) and (**I**) osteoclast length (OcL). All values are mean ± SEM for 4–6 mice per group.

Histomorphometry of the tibia following 5 vaginal applications of PEGLA (5 µg/g/application) to mated females (n = 2–4/group) showed no effect on trabecular bone volume, number or thickness (Figure 3A–D) or on osteoclast/osteoblast number and size (Figure 3E–I) when compared to PEGMSA-treated controls.

### PEGLA had no effect on the central nervous system

**Figure 4:**
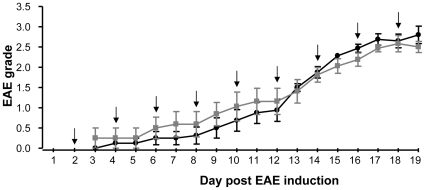
LIF antagonist treatment (↓) did not change EAE severity. EAE grades were recorded for MOG-induced EAE mice treated with PEG and LIF antagonist. •, PEG; ▪, LIF antagonist. All values are mean ± SEM for 8 mice per group.

PEGLA had no effect on EAE severity as determined by both motility scoring (Figure 4) and serum phosphorylated Neurofilament-H (pNF-H) levels 19 days after EAE induction (PEGLA: 3.9±1.4; PEG: 4.7±1.0 ng/ml; *P*>0.05). Serum pNF-H levels were highly correlated with mouse EAE clinical scores (Pearson correlation coefficient, *r* = 0.735).

### PEGLA tissue localisation and tissue half-life

To identify other potential tissue targets of PEGLA, we traced ^125^I-PEGLA or control, ^125^I-PEGBSA, following both IP injection and vaginal application.

#### PEGLA localised to the liver, ovary, oviduct, spleen and thyroid following IP injection

**Figure 5:**
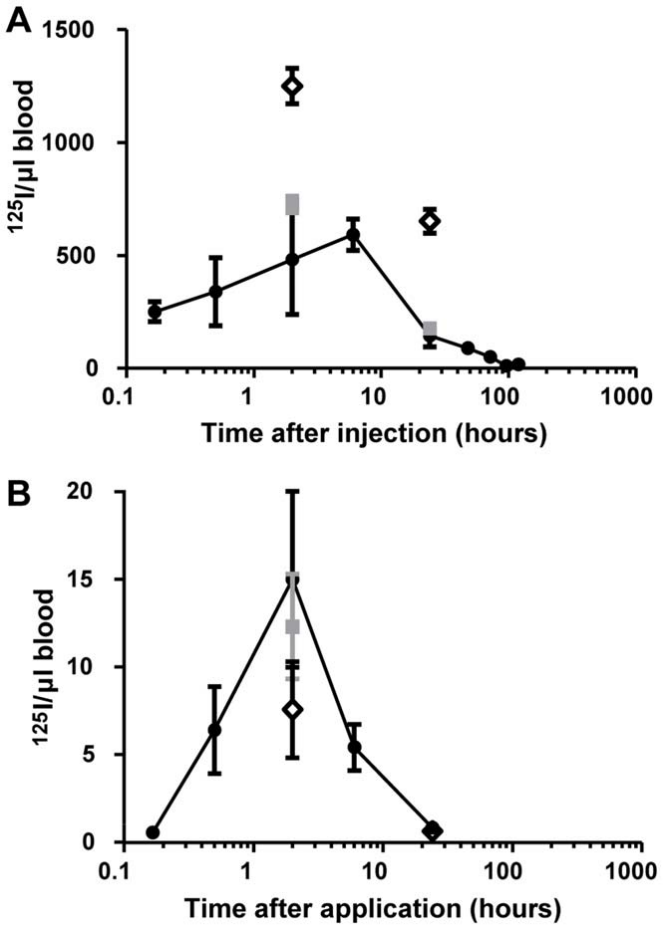
Circulating ^125^I following both IP and vaginal application. **A.** Circulating ^125^I in the 120 h following IP injection. **B.** Circulating ^125^I in the 120 h following vaginal application. •, ^125^I-PEGLA non-mated; ▪, ^125^I-PEGLA mated; ◊, ^125^I-PEGBSA non-mated. *, significant difference (*P*<0.05) to ◊.

^125^I-PEGLA was detected in blood from 10 min to 72 h after injection (Figure 5A). The concentration of ^125^I-PEGLA in blood peaked at 6 h post-injection (Figure 5A). The period of blood retention of ^125^I was equivalent to the retention of ^125^I-PEGLA (Figure S3A) although counts of ^125^I-PEGLA were between 53 and 89% of total ^125^I (Figure S3B), suggesting that up to 47% of total ^125^I in blood was free iodine.

After 24 h, most of the ^125^I had accumulated in the thyroid (Figure S4). In most tissues ^125^I accumulation peaked between 10 min and 6 h and fell to low levels by 24 h (Figure S4).

**Figure 6:**
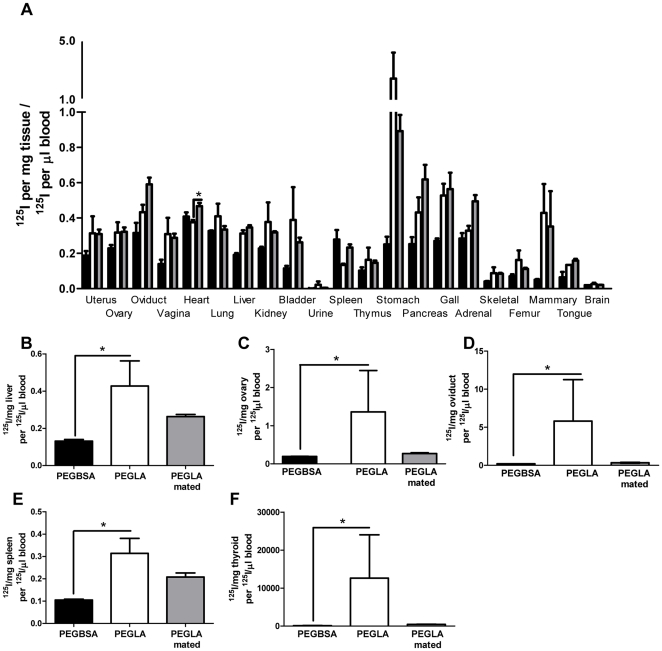
Tissue accumulation of ^125^I-PEGLA and ^125^I-PEGBSA following IP injection in non-mated (PEGLA: black; PEGBSA: white) and mated (PEGLA: grey) females. The counts given are normalised to the ^125^I counts in total blood (counts/µl). **A.** 2 h post-injection; **B.** Liver at 24 h post-injection; **C.** Ovary at 24 h post-injection; **D.** Oviduct at 24 h post-injection; **E.** Spleen at 24 h post-injection; **F.** Thyroid at 24 h post-injection. *, significant difference between treatment groups (p<0.05).

To determine whether the tissue accumulation of PEGLA was specific (ie. receptor bound) or non-specific (due to protein kinetics related to size) we compared accumulation of ^125^I-PEGLA with accumulation of a similarly sized control, ^125^I-PEGBSA. In non-mated females there was no difference in accumulation of ^125^I-PEGLA or control in any tissue at 2 h post-injection (Figure 6A; Figure S5A), but at 24 h post-injection (Figure S5B) ^125^I-PEGLA counts were significantly higher than ^125^I-PEGBSA counts in the liver (*P* = 0.0258; Figure 6B), ovary (*P* = 0.0429; Figure 6C), oviduct (*P* = 0.0343; Figure 6D), spleen (*P* = 0.0181; Figure 6E) and thyroid (*P* = 0.0258; Figure 6F) suggesting that PEGLA localised to and may be acting on these tissues. At 2 h post-injection, ^125^I-PEGLA accumulation in the heart of mated females was significantly higher than in the heart of non-mated females (*P* = 0.0349; Figure 6A).

#### Vaginal application decreased the half-life and tissue localisation of PEGLA

^125^I-PEGLA was detected in blood from 30 min to 24 h after vaginal application (Figure 5B). The concentration of ^125^I-PEGLA in blood peaked at 2 h after application (Figure 5B). The period of blood retention of ^125^I was equivalent to the retention of ^125^I-PEGLA (Figure S3C), although counts of ^125^I-PEGLA were between 37% and 72% of total ^125^I counts (Figure S3D), suggesting that up to 63% of total ^125^I counts in blood was free iodine.

After 24 h, the majority of ^125^I-PEGLA had accumulated in the thyroid (Figure S5). In all other tissues (except vagina & urine), ^125^I-PEGLA accumulation peaked at 2 h after application (Figure S6). Vaginal accumulation of ^125^I-PEGLA peaked 30 min following application (Figure S6) and urine accumulation of ^125^I-PEGLA peaked at 10 min and 2 h after application (Figure S6), suggesting a large amount of ^125^I-PEGLA was excreted almost immediately after application. No specific (receptor bound) accumulation of ^125^I-PEGLA was found in any tissue at 2 h or 24 h after vaginal application (Figure S7).

### ^125^I-PEGLA accumulated more quickly following IP injection than vaginal administration

To compare ^125^I accumulation between the two delivery routes the tissue ^125^I counts were normalised to the total ^125^I counts administered. At 10 min after administration, accumulation of ^125^I in most tissues was significantly lower following vaginal administration compared to IP injection (*P* = 0.0286 for all; Figure S8), except in the pancreas and urine, where ^125^I accumulation was equivalent between the two delivery methods, and in the vagina where ^125^I accumulation was higher (*P* = 0.0286; Figure S8). At all time-points after 10 min, except in the bladder (30 min, *P* = 0.0052) ^125^I accumulation was equivalent following IP injection and vaginal administration in all tissues examined.

## Discussion

Here we showed that vaginal administration of PEGLA was effective as a contraceptive in mice and also that this method of administration abolished the adverse effects of PEGLA on bone which were observed following IP injection. Further, we showed that PEGLA had no detectable effect on the CNS based on EAE induction. This is the first study to demonstrate that a large, PEGylated protein could be absorbed from the vagina and was active in the reproductive tract. The physiochemical properties of drugs are critical for vaginal absorption: the vagina is considered permeable to low molecular weight, lipophilic and hydrophilic proteins [41]. PEGLA is a large protein (∼60 kDa) but PEGylation increases water solubility of molecules. This study suggests that the vagina may be a suitable route of administration of a LIF antagonist for contraceptive purposes in women, providing an option for a non-steroidal contraceptive that can be combined with a microbicidal drug for inhibition of STIs, including HIV.

Identifying non-uterine targets of PEGLA is critical for its future contraceptive development. PEGLA had no effect on the CNS and importantly, although bone was identified as a non-uterine target of PEGLA following IP injection, no effect on bone was observed following vaginal application, suggesting that local administration limited the systemic effects of PEGLA. Only following IP injection was the specific accumulation of PEGLA in tissue observed: in the liver, ovary, oviduct, spleen and thyroid in non-mated females and in the heart of mated females. The long-term effect of PEGLA on these target tissues should be carefully evaluated in future studies, however, the elimination of the bone effect and the lack of specific tissue accumulation of PEGLA following vaginal administration suggests that local delivery may minimise any systemic and non-uterine effects of PEGLA.

This study also highlights the potential of vaginal administration for therapies targeting the female reproductive tract. Vaginally administered PEGLA localised to the ovary and oviduct more rapidly and at higher concentrations compared to other organs. Vaginal administration eliminated non-target, systemic effects of PEGLA and the dose of PEGLA required to inhibit implantation following vaginal administration was considerably lower than following IP injection. This suggests that non-target, systemic effects of drugs would be significantly diminished by vaginal administration. Currently, only small molecules such as steroid hormones are administered vaginally. We suggest that larger molecules, which have potential to treat pathologies such as endometrial or ovarian cancer, ectopic pregnancies or endometriosis can also be administered vaginally.

IP injection of PEGLA resulted in increased osteoclast number and size, which is consistent with the reported phenotypes of the LIF^−/−^, CT-1^−/−^ and LIFR^−/−^ mice [21], [22], [23]. In contrast, whilst no change in osteoblast generation was reported in the LIF^−/−^ or LIFR^−/−^ phenotypes [21], [22], we observed reduced osteoblast, osteoclast and osteoid surface in PEGLA treated mice and, only in mated females, increased trabecular bone volume. The inhibitory effect of a LIFR antagonist on osteoblast differentiation is consistent with reports that the LIFR ligands LIF and CT-1 stimulate osteoblast differentiation and bone formation [23], [42] and that OSM, acting through LIFR in mice, stimulates bone formation [43]. It must be noted that in this study, PEGylation reagent control females were pregnant, whereas PEGLA treated females had failure of implantation and were pseudo-pregnant at the time of bone collection. Thus, bone remodelling could differ due to pregnancy status rather than PEGLA treatment. Non-mated females however showed a similar response to PEGLA treatment, indicating that PEGLA inhibited bone remodelling regardless of pregnancy status, although the magnitude of the effect may still be modulated by pregnancy status.

A possible interaction of mating and pregnancy on bone metabolism was identified in this study. To date, most studies on the effects of mating and pregnancy on bone metabolism have focused on the late stages of pregnancy, when calcium is transferred to the fetus to allow mineralization of the developing skeleton [44]. Surprisingly, rapidly decreased trabecular bone volume and osteoblast surface and increased osteoclast surface following mating was observed. Whether such rapid bone depletion during early gestation occurs in species other than mice is unknown and requires investigation.

While a low level of bone remodelling in growing mice increases trabecular bone volume [23], low bone remodelling in adult humans increases fracture risk [45]. This is an important consideration for long-term management of patients using PEGLA as a contraceptive. Currently, both the combined oral hormonal contraceptives (COC) and progestin-only contraceptives (POC) are associated with changed bone metabolism, including decreased bone turnover (COC, POC) and bone resorption (POC) [46]. By contrast, the progestin-releasing intrauterine device Mirena is not associated with reduced bone mineral density [47], likely because systemic levels of progestin are minimized by the local site of administration and the reduced dose required. Thus, local delivery of PEGLA in women could be expected to reduce effects on bone metabolism as was found in mice in the present study.

LIF and CNTF are produced in response to an autoimmune insult within the central nervous system, most likely by reactive astrocytes to limit immune-mediated demyelination [36]. In mice, therapeutic recombinant LIF injection ameliorates EAE severity [36] and systemic injection of neutralizing anti-LIF antibodies worsens EAE [48]. Here, PEGLA did not worsen EAE severity as measured by two separate experiments: scoring of motility, and serum pNF-H levels, which signify axonal injury. Very little ^125^I-PEGLA was detected in the brain, further supporting the suggestion that PEGLA did not enter the CNS, probably due to the large hydrodynamic volume of PEGylated LA [19]. This suggests that PEGLA is unlikely to affect LIF action in the CNS where it plays an important role in oligodendrocyte survival [36].

### Conclusion

There is unmet need for contraception worldwide, particularly in developing countries [49]. Sub-Saharan Africa has the highest global burden of HIV infection and the majority of new infections in sub-Saharan Africa are transmitted via heterosexual intercourse [50]. Vaginally administered ‘dual-role’ contraceptives that also block HIV infection are highly desirable [49]. PEGLA differs from current pharmacological contraceptives in that it is non-hormonal and acts to keep the uterus in a non-receptive state so that implantation can not occur [19]. This study demonstrated that vaginal administration of PEGLA inhibited implantation in mice and abolished the non-target effect on bone and that PEGLA did not enter the CNS. Future studies are required to demonstrate the contraceptive efficacy of PEGLA in non-human primates to progress this research to human clinical trials.

## Supporting Information

Figure S1 Inhibition of LIF-induced proliferation of Ba/F3 cells expressing human LIFR and IL6ST by PEGLA. The maximum possible value for cell number in this assay was 200.** •**, PEGLA (19); ▪, PEGLA (this study).
(JPG)

Figure S2 Localization of PEGLA and inhibition of implantation following vaginal application of PEGLA in CAPRISA 400 gel (1∶2 dilution) to mated mice. **A–D.** PEGLA localization in the uterine luminal (**A&C**) and glandular epithelium (**B&D**), at 3 h (**A&C**) and 24 h (**B&D**) following vaginal application PEGLA. Arrows indicate positive staining for PEGLA. Insert, negative control tissue incubated with IgG control at the same concentration as the primary antibody. Bars 100 µm. **E&F.** PEGLA localization in the vaginal epithelium 3 h after a single application (**a**) and following repeated applications (10×) of (**F**) PEGLA. Arrows indicate positive staining for PEGLA. Insert, negative control tissue incubated with IgG control at the same concentration as the primary antibody. Bars 100 µm.
(JPG)

Figure S3 **A.** Circulating protein bound (TCA-precipitable) ^125^I in the 120 h following IP injection. **B.** Percentage of total ^125^I counts in blood that are protein bound (TCA-precipitable) in the 120 h following IP injection. **C.** Circulating protein bound (TCA-precipitable) ^125^I in the 96 h following vaginal application. **D.** Percentage of total ^125^I counts in blood that are protein bound (TCA-precipitable) in the 96 h following vaginal application. •, ^125^I-PEGLA non-mated; ▪, ^125^I-PEGLA mated; ◊, ^125^I-PEGBSA non-mated. *, significant difference (*P*<0.05) to ◊.
(JPG)

Figure S4 Tissue accumulation of ^125^I in the 120 h following IP injection. •, ^125^I-PEGLA non-mated; ▪, ^125^I-PEGLA mated; ◊, ^125^I-PEGBSA non-mated.
(JPG)

Figure S5 Tissue accumulation of ^125^I-PEGLA and ^125^I-PEGBSA at 2 h (A) and 24 h (B) following IP injection. Thyroid accumulation not shown due to scale. There was no difference in thyroid accumulation between the 3 groups. ▪, ^125^I-PEGBSA non-mated; ▪, ^125^I-PEGLA non-mated; □ ^125^I-PEGLA mated. *, significant difference (*P*<0.05).
(JPG)

Figure S6 Tissue accumulation of 125I in the 24 h following vaginal application. •, ^125^I-PEGLA non-mated; ▪, ^125^I-PEGLA mated; ◊, ^125^I-PEGBSA non-mated.
(JPG)

Figure S7 Tissue accumulation of ^125^I-PEGLA and ^125^I-PEGBSA at 2 h (A) and 24 h (B) following vaginal application. Thyroid accumulation not shown due to scale. There was no difference in thyroid accumulation between the 3 groups. ▪, ^125^I-PEGBSA non-mated; ▪, ^125^I-PEGLA non-mated; □ ^125^I-PEGLA mated. *, significant difference (*P*<0.05).
(JPG)

Figure S8 Tissue accumulation of ^125^I-PEGLA normalized to administered dose in the 24 h following IP injection ▪ and vaginal application ▪ ^125^I-PEGLA counts have been normalised to the total ^125^I-PEGLA counts administered (%AD, % administered dose) to allow a comparison between the two delivery routes. *, significant difference (*P*<0.05).
(JPG)
