# Supplementary figures and images for: Vaginally Administered PEGylated LIF Antagonist Blocked Embryo Implantation and Eliminated Non-Target Effects on Bone in Mice
Source: PLoS One. 2011 May 18;6(5):e19665. doi: 10.1371/journal.pone.0019665 (PMC3097203; doi:10.1371/journal.pone.0019665)

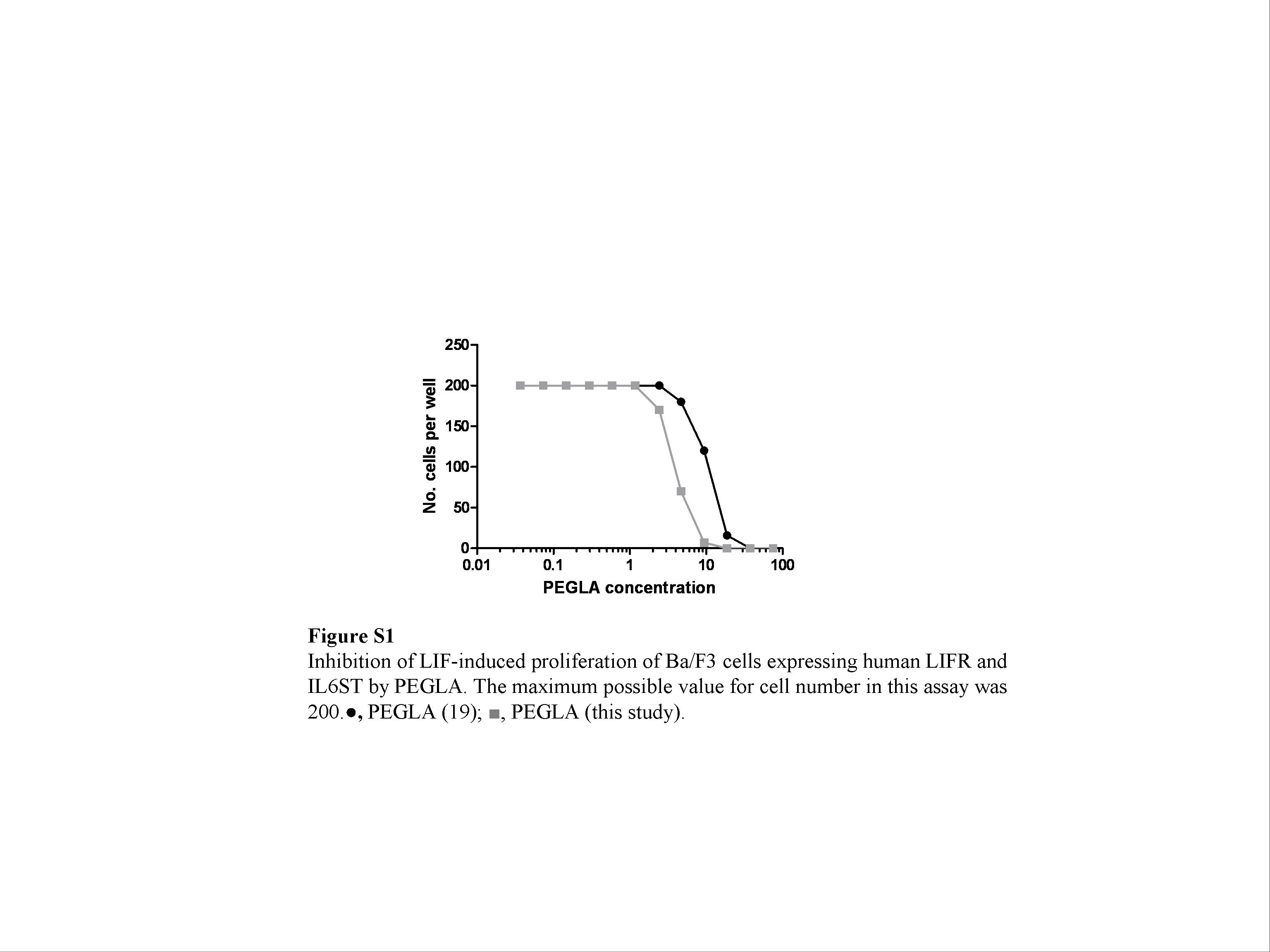

Supplement: Figure S1 — Inhibition of LIF-induced proliferation of Ba/F3 cells expressing human LIFR and IL6ST by PEGLA. The maximum possible value for cell number in this assay was 200. •, PEGLA (19); ▪, PEGLA (this study). (JPG) [file pone.0019665.s001.jpg]

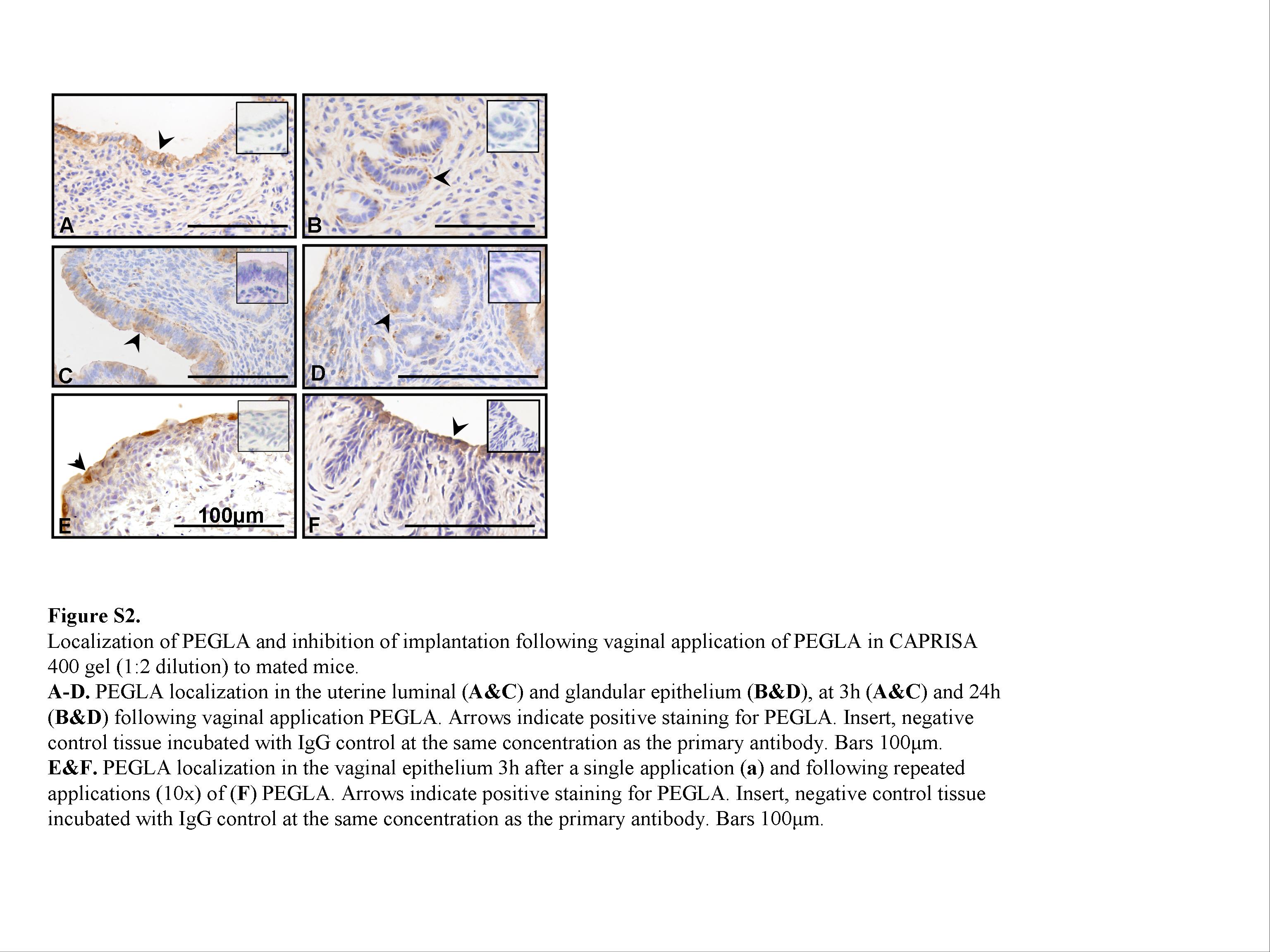

Supplement: Figure S2 — Localization of PEGLA and inhibition of implantation following vaginal application of PEGLA in CAPRISA 400 gel (1∶2 dilution) to mated mice. A–D. PEGLA localization in the uterine luminal (A&C) and glandular epithelium (B&D), at 3 h (A&C) and 24 h (B&D) following vaginal application PEGLA. Arrows indicate positive staining for PEGLA. Insert, negative control tissue incubated with IgG control at the same concentration as the primary antibody. Bars 100 µm. E&F. PEGLA localization in the vaginal epithelium 3 h after a single application (a) and following repeated applications (10×) of (F) PEGLA. Arrows indicate positive staining for PEGLA. Insert, negative control tissue incubated with IgG control at the same concentration as the primary antibody. Bars 100 µm. (JPG) [file pone.0019665.s002.jpg]

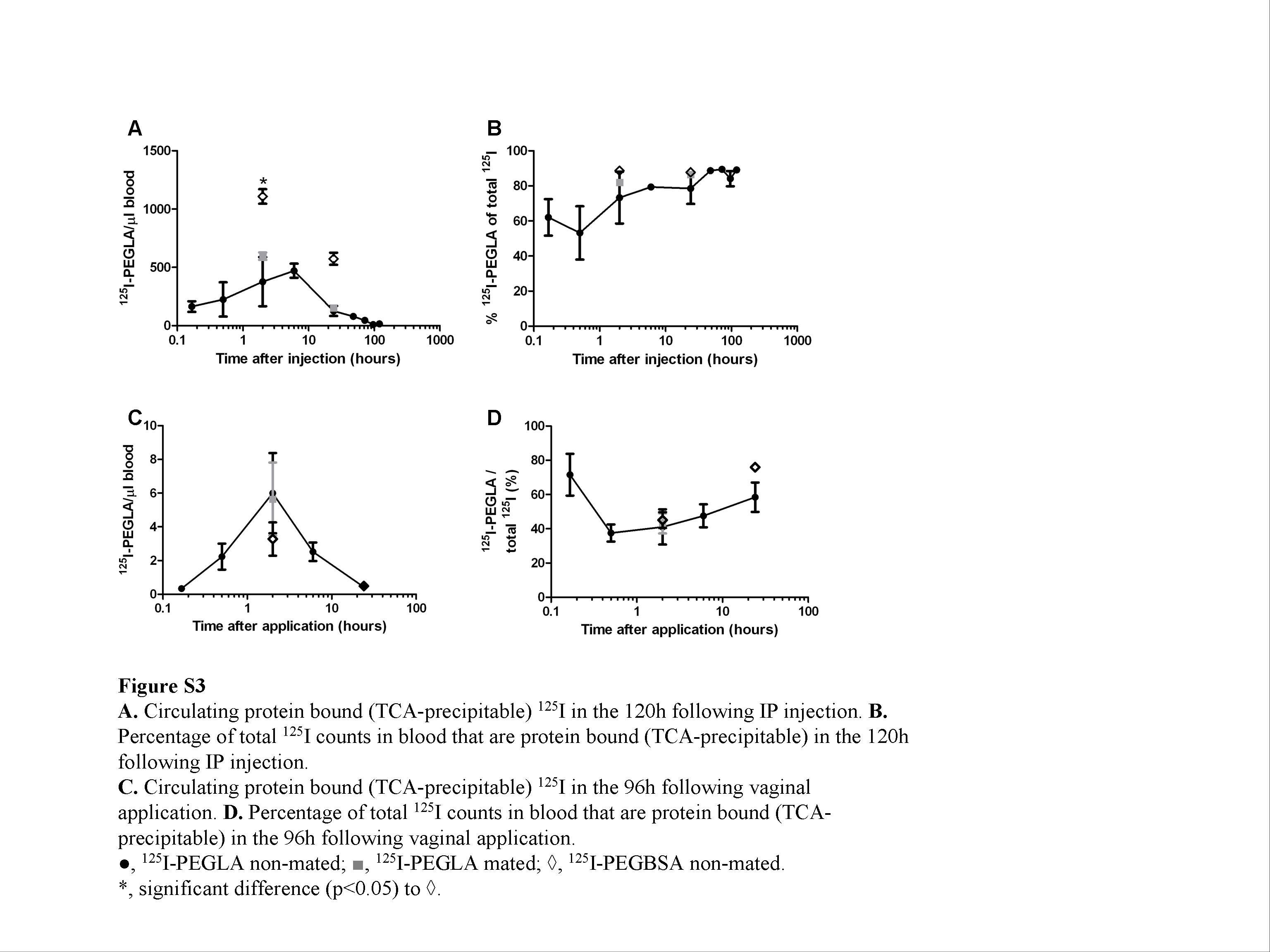

Supplement: Figure S3 — A. Circulating protein bound (TCA-precipitable) 125I in the 120 h following IP injection. B. Percentage of total 125I counts in blood that are protein bound (TCA-precipitable) in the 120 h following IP injection. C. Circulating protein bound (TCA-precipitable) 125I in the 96 h following vaginal application. D. Percentage of total 125I counts in blood that are protein bound (TCA-precipitable) in the 96 h following vaginal application. •, 125I-PEGLA non-mated; ▪, 125I-PEGLA mated; ◊, 125I-PEGBSA non-mated. *, significant difference (P<0.05) to ◊. (JPG) [file pone.0019665.s003.jpg]

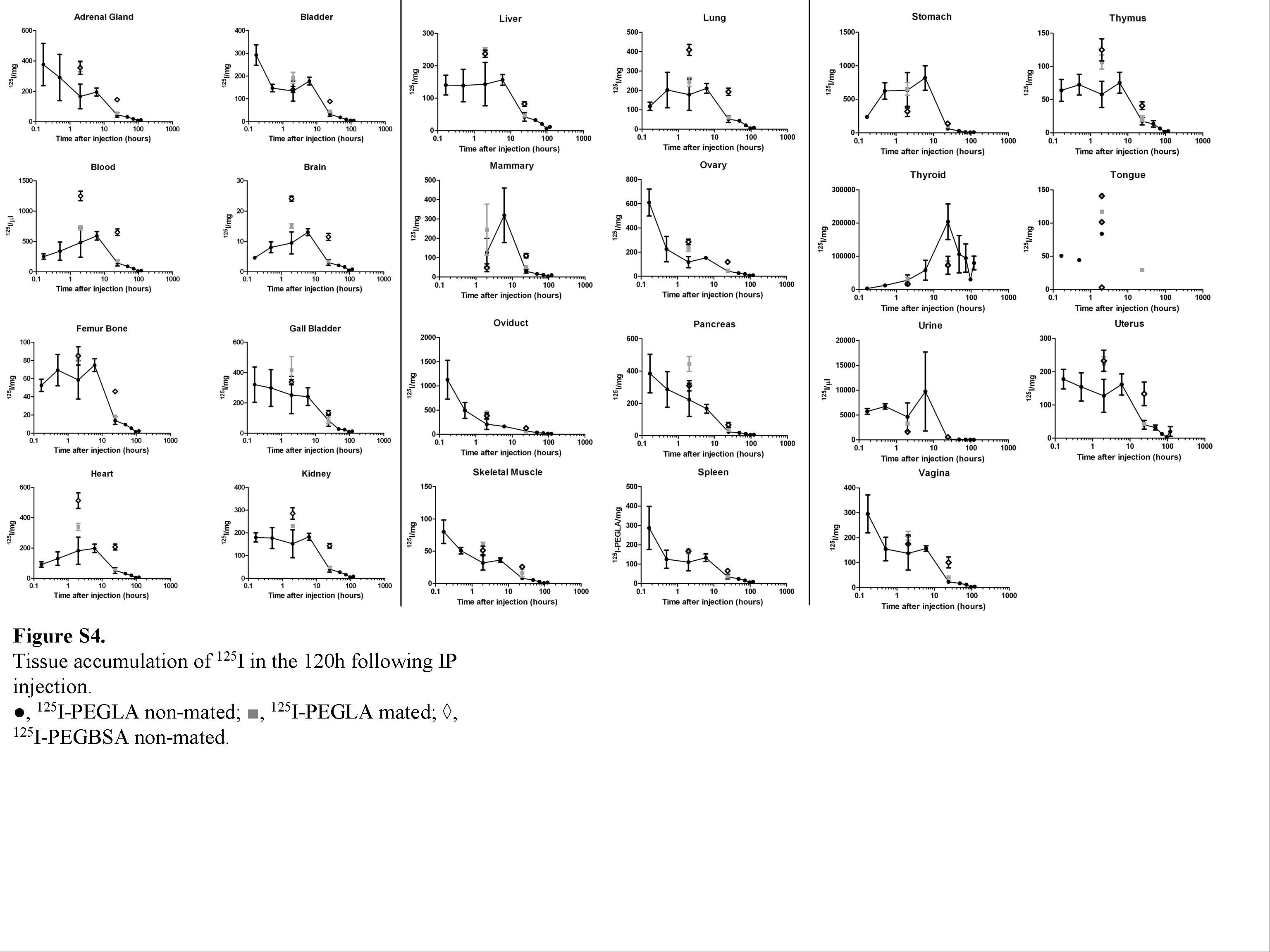

Supplement: Figure S4 — Tissue accumulation of 125I in the 120 h following IP injection. •, 125I-PEGLA non-mated; ▪, 125I-PEGLA mated; ◊, 125I-PEGBSA non-mated. (JPG) [file pone.0019665.s004.jpg]

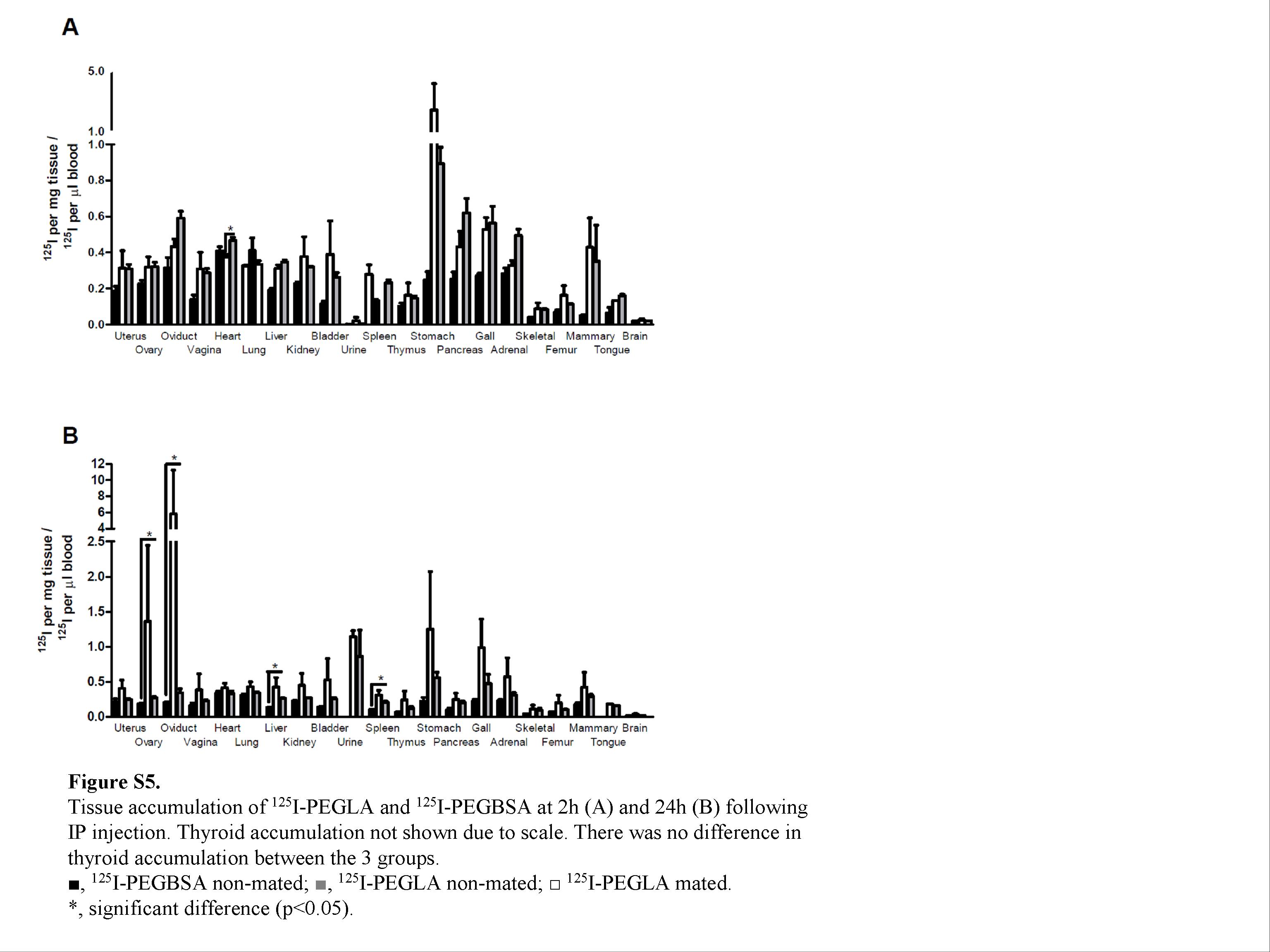

Supplement: Figure S5 — Tissue accumulation of 125I-PEGLA and 125I-PEGBSA at 2 h (A) and 24 h (B) following IP injection. Thyroid accumulation not shown due to scale. There was no difference in thyroid accumulation between the 3 groups. ▪, 125I-PEGBSA non-mated; ▪, 125I-PEGLA non-mated; □ 125I-PEGLA mated. *, significant difference (P<0.05). (JPG) [file pone.0019665.s005.jpg]

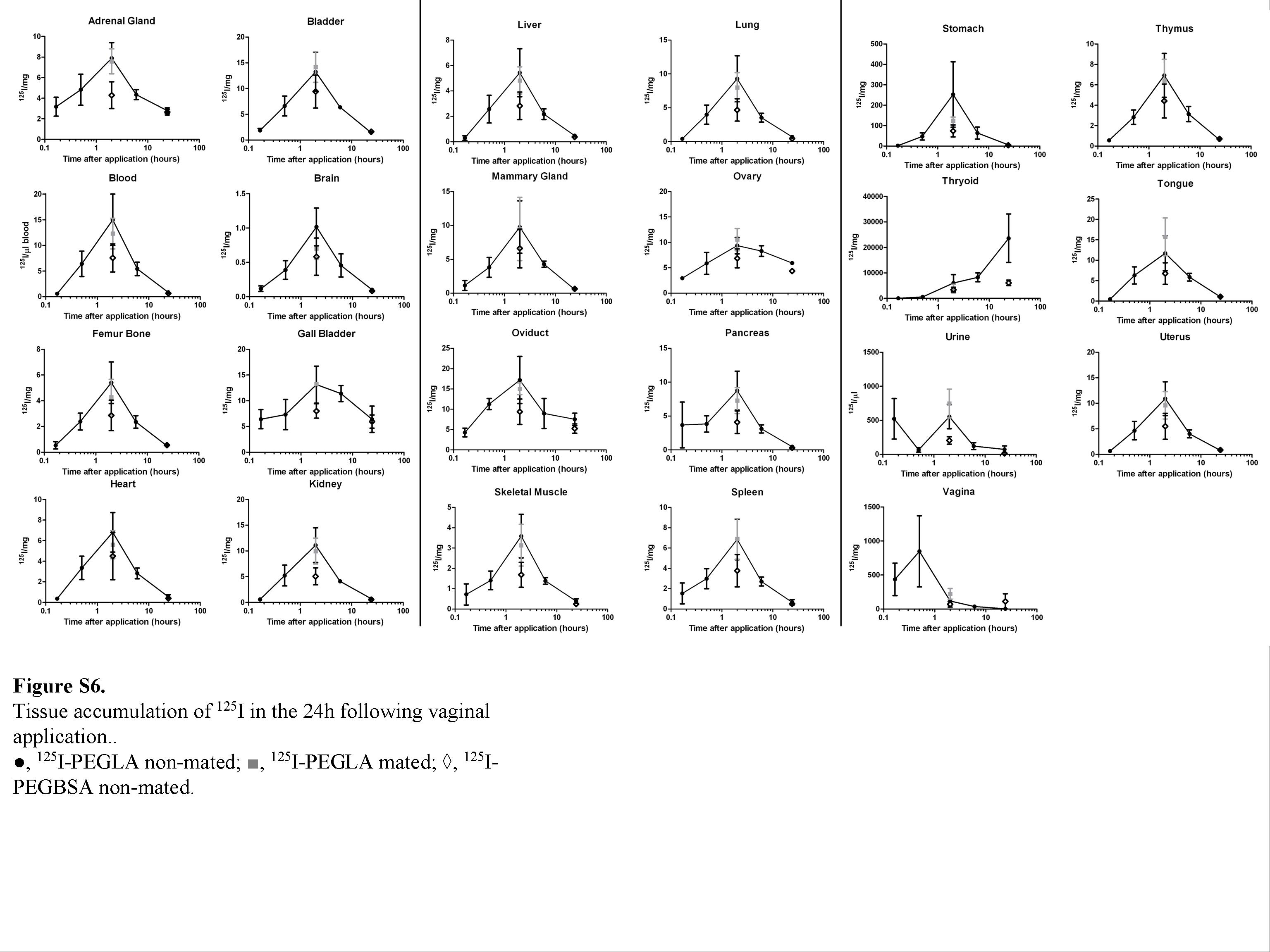

Supplement: Figure S6 — Tissue accumulation of 125I in the 24 h following vaginal application. •, 125I-PEGLA non-mated; ▪, 125I-PEGLA mated; ◊, 125I-PEGBSA non-mated. (JPG) [file pone.0019665.s006.jpg]

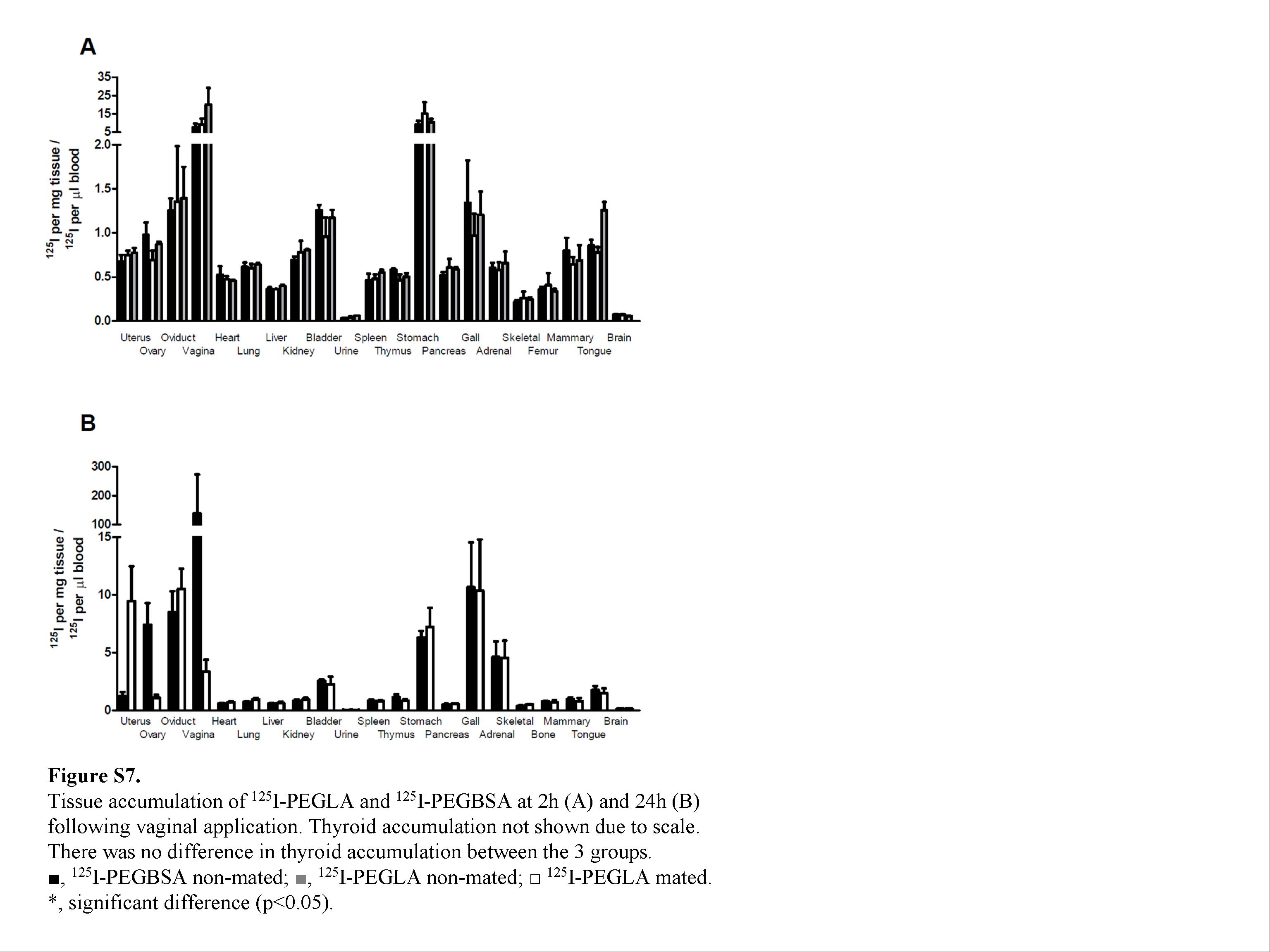

Supplement: Figure S7 — Tissue accumulation of 125I-PEGLA and 125I-PEGBSA at 2 h (A) and 24 h (B) following vaginal application. Thyroid accumulation not shown due to scale. There was no difference in thyroid accumulation between the 3 groups. ▪, 125I-PEGBSA non-mated; ▪, 125I-PEGLA non-mated; □ 125I-PEGLA mated. *, significant difference (P<0.05). (JPG) [file pone.0019665.s007.jpg]

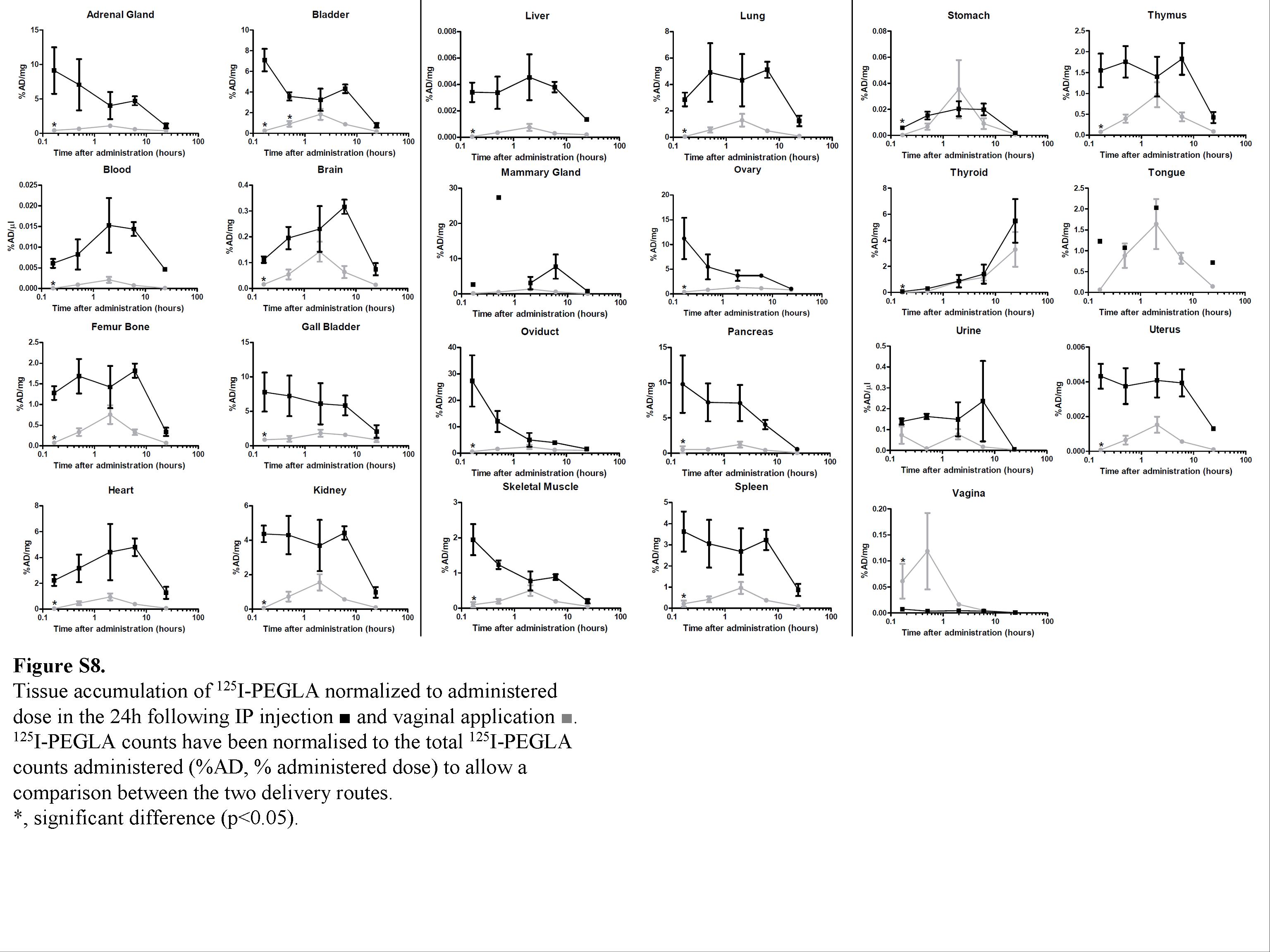

Supplement: Figure S8 — Tissue accumulation of 125I-PEGLA normalized to administered dose in the 24 h following IP injection ▪ and vaginal application ▪ 125I-PEGLA counts have been normalised to the total 125I-PEGLA counts administered (%AD, % administered dose) to allow a comparison between the two delivery routes. *, significant difference (P<0.05). (JPG) [file pone.0019665.s008.jpg]
